# Inference of human continental origin and admixture proportions using a highly discriminative ancestry informative 41-SNP panel

**DOI:** 10.1186/2041-2223-4-13

**Published:** 2013-07-01

**Authors:** Caroline M Nievergelt, Adam X Maihofer, Tatyana Shekhtman, Ondrej Libiger, Xudong Wang, Kenneth K Kidd, Judith R Kidd

**Affiliations:** 1 Department of Psychiatry, School of Medicine, University of San Diego California, La Jolla, CA, 92093, USA; 2 Department of Molecular and Experiment Medicine, The Scripps Research Institute, La Jolla, CA, 92037, USA; 3 Chongqing Medical University, Chongqing, Sichuan, China; 4 Genetics Dept, Yale University School of Medicine, New Haven, CT, 06520, USA

**Keywords:** Ancestry Informative Markers, Multiplex, Global Ancestry, Population Stratification, Admixture, AISNP, AIMs

## Abstract

**Background:**

Accurate determination of genetic ancestry is of high interest for many areas such as biomedical research, personal genomics and forensics. It remains an important topic in genetic association studies, as it has been shown that population stratification, if not appropriately considered, can lead to false-positive and -negative results. While large association studies typically extract ancestry information from available genome-wide SNP genotypes, many important clinical data sets on rare phenotypes and historical collections assembled before the GWAS area are in need of a feasible method (i.e., ease of genotyping, small number of markers) to infer the geographic origin and potential admixture of the study subjects. Here we report on the development, application and limitations of a small, multiplexable ancestry informative marker (AIM) panel of SNPs (or AISNP) developed specifically for this purpose.

**Results:**

Based on worldwide populations from the HGDP, a 41-AIM AISNP panel for multiplex application with the ABI SNPlex and a subset with 31 AIMs for the Sequenome iPLEX system were selected and found to be highly informative for inferring ancestry among the seven continental regions Africa, the Middle East, Europe, Central/South Asia, East Asia, the Americas and Oceania. The panel was found to be least informative for Eurasian populations, and additional AIMs for a higher resolution are suggested. A large reference set including over 4,000 subjects collected from 120 global populations was assembled to facilitate accurate ancestry determination. We show practical applications of this AIM panel, discuss its limitations for admixed individuals and suggest ways to incorporate ancestry information into genetic association studies.

**Conclusion:**

We demonstrated the utility of a small AISNP panel specifically developed to discern global ancestry. We believe that it will find wide application because of its feasibility and potential for a wide range of applications.

## Background

Characterization of human ancestry has been of interest for decades as information about population structure can provide novel insight into the human past and remains an important topic in the rapidly evolving biomedical field. For example, because genetic variants conferring risk to a particular disease may be geographically restricted because of evolutionary forces such as mutation, genetic drift, migration and natural selection, the assessment of the genetic background in individuals chosen for a study is crucial in genetic epidemiology [1].

While still a topic of controversy [2], there is ample evidence that self-reported race, as for example used in the US Census, can predict ancestral clusters in a population sample. However, it does not completely inform on how genetic variation is apportioned within and between racial groups, nor does information on race reveal the extent of admixture [2,3].

Especially in the context of mapping disease genes, more objective and accurate methods of defining homogenous populations for the investigation of specific population-disease associations are required. This is not only paramount for specific mapping approaches such as admixture mapping [4], but has also been recognized as a crucial prerequisite for genetic association studies, as the presence of undetected population structure can lead to both false-positive results and failures to detect genuine associations [5]. Furthermore, it has been shown that the consequences of population structure on association outcomes increase markedly with sample size, and even modest levels of population structure within population groups cannot safely be ignored in the large studies needed to detect typical genetic effects in common diseases [6].

In order to assess genetic background diversity, a large number of ancestry informative marker (AIM) panels have been developed for particular applications. Genome-wide panels for admixture mapping have been developed for Hispanic populations [7], African Americans [8] or three-way admixture in the Americas [9], and smaller AIM panels have been designed to discern ancestry at either the global level [10-12] or within specific populations such as the Native and Mexican Americans [13-15], Europeans [16-20] or African Americans [21,22]. In addition, genome-wide association studies (GWAS) are able to leverage ancestral information from the allele frequencies of the several thousand SNPs generated for whole-genome applications, alleviating the need for specific AIM panels [5].

However, determining ancestry and controlling for population structure is just as important in smaller genetic association studies. These include for example candidate gene studies involving only a few genetic markers, replication of GWAS findings, or consist of smaller, highly valuable collections of rare pathological phenotypes and historical collections with limited amounts of DNA. Genotyping these samples on large AIM panels or leveraging ancestry information from preexisting genotyping is often not practical or possible.

To address this specific need, we set out to develop a highly informative AIM panel that would allow us to infer a subject’s ancestral origin at the continental level and estimate admixture proportions among at least seven main geographic regions Africa, the Middle East, Europe, Central and South Asia, East Asia, Oceania and the Americas. The selection of such AIMs has to focus on SNPs with the largest allele frequency differences between the continental regions of interest to achieve the desired resolution at the continental level. Such high resolution is required because genetic diversity of human populations follows gradients or geographic clines within and among continents rather than specific clusters or clades [3,23,24].

We further aimed for the development of a feasible method to determine ancestry, as resources such as funding and available DNA are often limited for these applications. We therefore developed panels of AISNPs suitable for multiplex application on two commonly used platforms, the ABI SNPlex [25] and Sequenome iPLEX [26] systems. Additionally, all markers are also included on the Illumina HumanHap550 array, thus allowing for a combined analysis with studies genotyped on the Illumina whole-genome arrays.

Lastly, we specifically focused on the applicability of our panel to determine the ancestry of subjects from any of the worldwide geographic origins. To date, most research involving genetic association studies has focused on populations of European descent, where longer LD blocks require fewer genetic markers to be genotyped [27]. However, current gene-mapping efforts specifically request more global research, thus increasing the need for global AIM panels. Furthermore, global ancestry determination is especially important in clinical samples ascertained in specific geographic regions such as Southern California that are inhabited by individuals with very diverse and often heavily admixed ancestries.

Here we describe the development of AIM panels based on the well-studied global reference populations from the HGDP-CEPH [28], which include 52 geographically diverse populations collected from seven continental regions. We then greatly expanded the reference population set by genotyping the AIMs in over 2,000 additional subjects of known ancestry with the goal of achieving the most comprehensive global reference collection possible. We report on these efforts and describe highly discriminative ancestry informative 41- and 31-marker panels for multiplex applications.

## Methods

### Reference populations

AIM panels were developed based on the global reference populations from the HGDP-CEPH [28]. A total of 941 subjects including 52 populations from the standardized H952 subset were selected [29]. Based on the geographic origin of the samples, HGDP subjects were assigned to one of seven geographic or continental regions: Africa (*n* = 131), the Middle East (including the North African Moabites, *n* = 133), Europe (*n* = 158), Central/South Asia (CS Asia, *n* = 198), East Asia (E Asia, *n* = 229), the Americas (*n* = 64) and Oceania (*n* = 28) (Additional file 1: Table S1).

### AIM panel development

Genotypes of HGDP subjects from the Illumina 650Y SNP array are publicly available (http://hagsc.org/hgdp/files.html). We used Infocalc 1.1 [30] to calculate the marker informativeness (I_n) among the seven continental regions for each of the 644,195 autosomal markers. The mean informativeness of all markers was 0.0539, with a wide range of I_n = 0.0003-0.406. AIMs were selected according to the following criteria: being autosomal, unambiguous (AC, AG, TC, TG) and present on the Illumina Hap550 array (*n* = 547,458). Next, the top 5,000 markers with the highest I_n were chosen (I_n > 0.077) and, to reduce the correlation of markers, were subjected to LD pruning using PLINK [31] at a VIF = 1.5. The resulting pool of AIMs included 1,442 SNPs (Additional file 2: Table S2).

A small panel for multiplexing applications was developed by first choosing from the pool of 1,442 AIMs the top ten markers with the highest allele frequency differences (δ) between each of the 21 pairwise continental region comparisons. This set of 210 markers was then further reduced in an iterative way by considering multiplex genotyping requirements for the ABI SNPlex genotyping system [25] and Sequenome iPLEX system [26], leading to the final 41-AIM set for ABI SNPlex genotyping and the matching 31-AIM set for Sequenome iPLEX genotyping.

### Additional reference and test populations

To validate the AIM panels and increase the global coverage of the reference population set for downstream applications, we included two additional, very large data sets with worldwide populations: the International HapMap Project (http://hapmap.ncbi.nlm.nih.gov/; phase III release 2 and 3) standard set HAP1161 [32] included 931 subjects from 11 populations, and the Yale data set included 2,146 subjects from 57 populations [33]. The combined reference set included 4,018 unrelated subjects from 120 (partially overlapping) populations (Additional file 1: Table S1). These reference populations have been described previously [33], and geographic features such as latitude and longitude of these populations are presented in the allele frequency database ALFRED (http://alfred.med.yale.edu/) [34]. Genotypes of at least 40 of the 41 AIMs were available for all reference subjects.

Finally, to illustrate a practical application of the 41-AIM panel with our complete set of global reference populations, a contemporary population sample of 2,392 subjects ascertained in Southern California [35] was genotyped using the ABI SNPlex system. Ancestry was determined for all subjects with < 5% genotypes missing.

### Statistical analyses

Population structure and individual ancestry estimates were obtained using STRUCTURE v2.3.2.1. [36,37]. To assess the global informativeness of the 41-AIM panel in the original HGDP reference populations, five independent runs without prior population assignment were performed at K = 2 to K = 7, using 20,000 burn-in cycles and 20,000 MCMC replications under the admixture model. The “infer α” option with the same, uniform alpha for all populations was used under the λ = 1 option. All other parameters were set at default.

To further validate the 41-AIM panel, ancestry estimates of 3,077 independent subjects of known ancestry from 68 global populations (reference set 2) were determined at k = 7 using the above STRUCTURE parameters, but now including prior population information of the HGDP reference set. Allele frequencies were updated using only individuals with population information at a migration prior of 0.05. Graphs were plotted using DISTRUCT v1.1 [38].

CLUMPP v1.1.2 [39] was used to evaluate different replicates of STRUCTURE runs. To assign a subject to a specific cluster, we applied cutoffs of >85% and >50% cluster membership, respectively. These criteria were selected to facilitate a comparison with Seldin's 93-AIM panel [10]. Finally, to validate the AIM panels, the percentage of subjects that clustered correctly compared to the known geographic origin was calculated.

Population structure was further analyzed using principal component analysis (PCA) implemented in the EIGENSTRAT software [40] and multi-dimensional scaling (MDS) as implemented in PLINK. All other calculations were performed in R v2.15.0.

As a measure of informativeness of the different AIM panels at the population level, we calculated F_ST_, a genetic distance measure for inter-population differentiation compared to intra-population variation. Significance of pairwise F_ST_s was established using 10,000 permutations. A Mantel test was used to correlate the F_ST_ matrices based on the 41-AIM and 31-AIM panels. Calculations were performed in ARLEQUIN 3.5 [41].

To investigate the informativeness of the AIM panels in detecting admixture at the individual level, subjects from two admixed populations of the Southern California test population (self-reported African Americans and self-reported Hispanic White and Native Americans) were selected. These subjects were subjected to the Illumina HumanOmniExpressExome array, and individual ancestry estimates were determined with a second, independent approach (see [42] for details). In brief, we used over 10,000 GWAS-derived SNPs, a set of 2,513 (partly overlapping) reference individuals and a two-step analysis approach implemented in ADMIXTURE [43]. Individual admixture estimates based on the GWAS-derived panel were then compared to the admixture estimates based on the 41- and 31-AIM panels for these two admixed populations (see above).

## Results

### Characterization of small AIM panels to determine continental ancestry

Fifty-two global populations from the HGDP-CEPH panel [28] were used to select AIMs optimized for the determination of continental ancestry. We developed a small 41-AIM panel specifically for multiplex application on the ABI system from a pre-selected pool of 1,442 highly informative AIMs (Additional file 1: Table S1). The panel was further reduced to 31 AIMs for application on the Sequenome iPLEX system.

Table 1 shows the informativeness (I_n) and pairwise allele frequency differences (δ) among the seven continental regions for each of the 41 AIMs. I_n ranges from 0.08 - 0.41 with a high mean of 0.23. The largest I_n and largest δ for each of the 21 continental comparisons are indicated in bold, highlighting the strength of a marker to distinguish between specific different global origins. Most continental comparisons included several markers with very high δ of >0.8. The smallest allele frequency differences were found for comparisons of regions within Eurasia where the top markers showed δ in the range of 0.4, indicating limited power to accurately distinguish subjects from Europe, the Middle East and Central/South Asia from each other.

**Table 1:** Informativeness (I_n) and allele frequency differences (δ) between seven continental regions for SNPs on the 41-AIM panel

**Continental regions: 1 = Africa, 2 = the Americas, 3 = Central/South Asia, 4 = East Asia, 5 = Europe, 6 = the Middle East, 7 = Oceania**																						
**SNP**	**I_n**	**1-2**	**1-3**	**1-4**	**1-5**	**1-6**	**1-7**	**2-3**	**2-4**	**2-5**	**2-6**	**2-7**	**3-4**	**3-5**	**3-6**	**3-7**	**4-5**	**4-6**	**4-7**	**5-6**	**5-7**	**6-7**
rs1834640	**0.406**	0.367	**0.838**	0.090	**0.945**	**0.918**	0.004	0.471	0.277	0.578	0.551	0.371	**0.749**	0.107	0.080	**0.842**	**0.855**	**0.828**	0.093	0.027	**0.949**	**0.921**
rs9809818*	**0.364**	**0.811**	0.355	**0.822**	**0.**057	0.079	**0.972**	0.455	0.012	0.754	0.731	0.162	0.467	0.299	0.276	0.617	0.765	0.743	0.150	0.023	**0.916**	**0.893**
rs310644	**0.352**	**0.909**	0.741	**0.925**	**0.899**	**0.812**	0.063	0.168	0.016	0.010	0.097	**0.846**	0.184	0.158	0.071	0.678	0.026	0.114	**0.862**	0.087	**0.836**	0.749
rs1834619*	**0.334**	**0.953**	0.280	0.723	0.073	0.028	0.714	0.673	0.230	**0.880**	**0.926**	0.239	0.443	0.207	0.252	0.434	0.650	0.695	0.008	0.045	0.642	0.687
rs1572018*	**0.320**	**0.930**	0.718	0.340	**0.877**	0.724	0.036	0.212	0.590	0.053	0.206	0.**894**	0.378	0.159	0.006	0.682	0.537	0.384	0.304	0.153	**0**.**841**	0.688
rs7226659*	0.**318**	0.415	0.055	0.575	0.002	0.019	**0.950**	0.360	0.160	0.417	0.396	0.535	0.520	0.057	0.036	**0.894**	0.577	0.556	0.375	0.021	**0.952**	**0.931**
rs260714*	0.**302**	0.064	0.589	0.107	0.705	0.601	0.164	0.653	0.043	0.769	0.665	0.099	**0.696**	**0.**116	0.012	0.752	**0.812**	0.708	0.056	0.104	**0.868**	0.764
rs4918664	0.287	**0.855**	0.285	**0.869**	0.147	0.038	0.174	0.569	0.014	0.708	**0.816**	0.681	0.583	0.138	0.247	0.111	0.722	**0.830**	0.695	0.109	0.027	0.136
rs4471745	0.284	0.097	0.003	0.023	0.050	0.104	**0.869**	0.094	0.120	0.048	0.006	**0.967**	0.025	0.047	0.101	**0.872**	0.072	0.126	**0.847**	0.054	**0.919**	**0.973**
rs11725412	0.272	0.704	0.047	0.432	0.186	0.243	0.421	0.751	0.272	**0.890**	**0.947**	0.284	0.479	0.138	0.196	0.468	0.617	0.675	0.011	0.058	0.606	0.664
rs3098610	0.271	0.761	0.328	**0.849**	0.265	0.399	**0.980**	0.433	0.088	0.496	0.362	0.219	0.521	0.063	0.071	0.652	0.584	0.450	0.131	0.134	0.715	0.581
rs4664511	0.268	0.568	0.143	0.502	0.043	0.090	**0.833**	0.424	0.066	0.611	0.658	0.266	0.358	0.187	0.233	0.690	0.545	0.591	0.332	0.047	**0.877**	**0.923**
rs10079352	0.267	**0.975**	.0540	**0.957**	0.459	0.312	0.419	0.434	0.017	0.516	0.662	0.556	0.417	0.082	0.228	0.121	0.499	0.645	0.539	0.146	0.040	0.107
rs2166624	0.262	**0.977**	0.323	0.430	0.383	0.224	0.000	0.654	0.546	0.594	0.753	**0.977**	0.108	0.060	0.099	0.323	0.047	0.206	0.430	0.159	0.383	0.224
rs12498138	0.247	**0.906**	0.070	0.090	0.060	0.040	0.089	**0.836**	**0.817**	**0.846**	**0.866**	**0.817**	0.020	0.010	0.030	0.019	0.029	0.050	0.000	0.020	0.029	0.049
rs7251928	0.245	0.966	0.778	0.942	0.741	0.662	0.787	0.188	0.024	0.225	0.304	0.179	0.164	0.037	0.116	0.009	0.201	0.280	0.155	0.079	0.046	0.125
rs6990312	0.244	0.457	0.516	0.787	0.621	0.505	0.166	0.060	0.330	0.164	0.048	0.623	0.271	0.104	0.012	0.683	0.166	0.282	**0**.**953**	0.116	0.787	0.671
rs3823159	0.242	0.477	**0.859**	0.745	**0.904**	**0.815**	0.371	0.382	0.268	0.427	0.338	0.106	0.114	0.044	0.045	0.488	0.158	0.070	0.374	0.089	0.533	0.444
rs2024566	0.236	**0.903**	0.257	0.012	0.218	0.239	0.056	0.647	**0.891**	0.685	0.664	**0.959**	0.245	0.039	0.017	0.312	0.206	0.228	0.067	0.022	0.273	0.295
rs7722456	0.235	0.362	0.268	0.351	0.175	0.178	0.569	0.094	0.011	0.187	0.184	**0.931**	0.084	0.093	0.089	**0**.**836**	0.176	0.173	**0.920**	0.003	0.744	0.747
rs9880567	0.232	0.567	0.326	0.441	0.307	0.353	0.402	0.241	0.126	0.260	0.214	**0.969**	0.115	0.019	0.027	0.728	0.134	0.088	**0.843**	0.046	0.709	0.755
rs842639	0.231	0.706	0.340	0.603	0.014	0.062	0.674	0.366	0.103	0.692	0.768	0.032	0.263	0.326	**0.402**	0.334	0.589	0.665	0.071	0.076	0.660	0.735
rs10497191	0.230	**0.883**	**0.858**	**0.893**	**0.822**	0.768	**0.889**	0.024	0.010	0.061	0.115	0.007	0.035	0.037	0.091	0.031	0.072	0.126	0.004	0.054	0.068	0.122
rs734241	0.227	**0.899**	0.211	0.311	0.003	0.035	0.464	0.688	0.589	**0.896**	**0.864**	0.435	0.100	0.208	0.176	0.253	0.308	0.276	0.153	0.032	0.461	0.429
rs735480*	0.224	0.459	0.755	0.597	**0.895**	**0.815**	0.205	0.296	0.138	0.436	0.356	0.255	0.158	0.140	0.060	0.551	0.298	0.218	0.393	0.080	0.691	0.611
rs2593595	0.218	0.615	0.688	**0.805**	0.753	0.683	**0.990**	0.073	0.189	0.138	0.068	0.375	0.117	0.065	0.004	0.303	0.052	0.121	0.186	0.069	0.237	0.307
rs2717329	0.213	0.762	0.603	0.710	0.759	0.520	**0.980**	0.159	0.052	0.003	0.241	0.219	0.107	0.156	0.083	0.378	0.049	0.189	0.271	0.239	0.222	0.460
rs10961366*	0.213	0.019	0.254	0.023	0.254	0.278	0.623	0.235	0.042	0.235	0.259	0.642	0.277	0.000	0.024	**0.876**	0.277	0.301	0.600	0.024	**0.877**	**0.900**
rs1557553	0.207	**0.887**	0.080	0.273	0.085	0.045	0.195	**0.806**	0.614	**0.802**	**0.842**	0.692	0.193	0.004	0.036	0.114	0.188	0.228	0.078	0.040	0.110	0.150
rs4741658*	0.202	0.742	0.458	0.774	0.225	0.117	0.630	0.285	0.032	0.518	0.626	0.113	0.317	0.233	0.341	0.172	0.549	0.658	0.144	0.108	0.405	0.513
rs2196051	0.202	0.012	0.228	0.002	0.645	0.493	0.020	0.216	0.014	0.633	0.481	0.031	0.230	**0.417**	0.265	0.248	0.647	0.495	0.018	0.152	0.665	0.512
rs6737672	0.199	**0.977**	0.585	0.620	0.516	0.561	0.321	0.392	0.357	0.461	0.415	0.655	0.035	0.069	0.024	0.264	0.104	0.059	0.299	0.046	0.194	0.240
rs10877030	0.185	0.472	0.082	0.231	0.197	0.318	0.460	0.390	0.241	0.669	0.790	0.012	0.149	0.279	**0.400**	0.378	0.428	0.549	0.229	0.122	0.657	0.779
rs7837234	0.162	0.113	0.003	0.271	0.039	0.044	0.619	0.110	0.158	0.152	0.069	0.732	0.268	0.042	0.041	0.622	0.310	0.227	**0.890**	0.083	0.580	0.663
rs4907251	0.157	**0.843**	0.550	0.528	0.205	0.242	0.475	0.292	0.315	0.638	0.600	0.367	0.023	0.346	0.308	0.075	0.323	0.286	0.052	0.038	0.271	0.233
rs1863086	0.149	0.658	0.133	0.214	0.255	0.116	0.310	0.525	**0.872**	0.403	0.542	0.348	0.347	0.122	0.017	0.177	0.469	0.330	0.524	0.139	0.055	0.194
rs310362*	0.147	0.763	0.476	0.533	0.627	0.279	0.751	0.287	0.229	0.136	0.484	0.011	0.058	0.151	0.197	0.276	0.094	0.255	0.218	**0.349**	0.124	0.473
rs4705360	0.139	0.701	0.300	0.397	0.047	0.011	0.419	0.401	0.304	0.654	0.712	0.282	0.097	0.253	0.312	0.119	0.350	0.409	0.021	0.058	0.372	0.430
rs4833103	0.110	0.016	0.038	0.009	0.449	0.104	0.000	0.022	0.007	0.434	0.089	0.016	0.029	**0.412**	0.067	0.038	0.441	0.096	0.009	0.345	0.449	0.104
rs359955*	0.090	0.455	0.533	0.613	0.639	0.298	0.410	0.078	0.158	0.183	0.157	0.046	0.080	0.106	0.235	0.124	0.026	0.315	0.204	0.341	0.229	0.111
rs12878166	0.078	0.137	0.220	0.413	0.098	0.259	0.038	0.356	0.549	0.039	0.396	0.174	0.193	0.317	0.039	0.182	0.510	0.154	0.375	**0.356**	0.135	0.221

*Not on the 31-AIM panel.

The AIM panels were further characterized by calculating F_ST_[41] as a measure of the panel’s relative strength to distinguish the seven geographic regions. Table 2 shows the genetic distance between the continental regions when using the 41-AIM (lower diagonal) and 31-AIM panel (upper diagonal), respectively. Inter-continent differentiation was based on allele frequencies from 51 HGDP populations; the atypical North African Mozabites were excluded here.

**Table 2:** **Pairwise F**_**ST **_**values among the seven continental regions calculated based on allele frequencies of 41 AIMs (below diagonal) and 31 AIMs (above diagonal)**

	**Africa**	**Middle East**	**Europe**	**CS Asia**	**East Asia**	**Americas**	**Oceania**
**Africa**		0.439	0.456	0.401	0.581	0.723	0.594
**Middle East**	0.457		0.043	0.076	0.365	0.535	0.501
**Europe**	0.498	0.054		0.087	0.358	0.496	0.479
**CS Asia**	0.417	0.080	0.086		0.206	0.369	0.372
**East Asia**	0.564	0.395	0.391	0.232		0.373	0.466
**Americas**	0.712	0.531	0.501	0.353	0.305		0.617
**Oceania**	0.632	0.552	0.543	0.411	0.398	0.555	

All FST values are significant at *p* < 0.0001.

In general, we found high F_ST_ values distinguishing the African, East Asian, American and Oceanian regions. As expected, the lower F_ST_ values among Europe, the Middle East and Central/South Asia reflect the I_n and δ found for the single markers. When comparing the F_ST_ values of the full 41-AIM panel with the reduced 31-AIM panel, no significant differences were found (Wilcoxon signed rank test, *n* = 21 paired comparisons, *p* > 0.38). In addition, a comparison of all pairwise F_ST_ values among the 52 populations showed a highly significant correlation among the F_ST_ values calculated based on the 41-AIM panel and the 31-AIM panel (Mantel test, *r* = 0.987, *p* < 0.001), further indicating no significant loss of power to discern global ancestry in the smaller panel.

**Figure 1:**
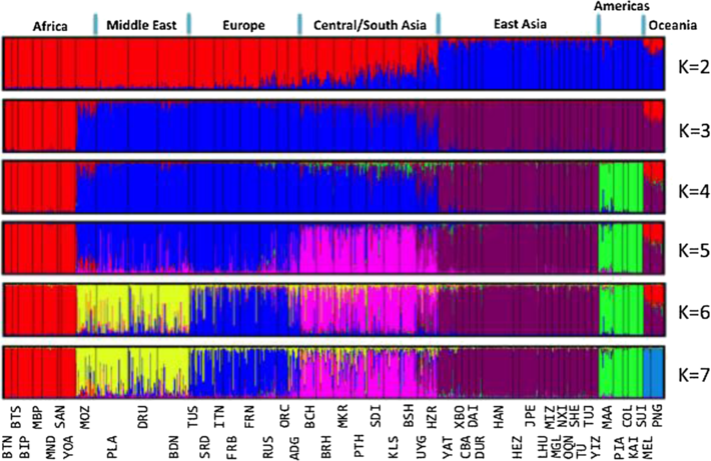
Inferred population structure of the HGDP subjects based on the 41-AIM panel with clusters ranging from K2 - K7.

Lastly, the population structure of the HGDP was analyzed using STRUCTURE. To facilitate a comparison with previous studies (e.g., [10,12,24,33,44]), we used similar model parameters without prior information about individual sampling locations. Figure 1 shows the most typical patterns with the highest likelihood from each of 20 independent runs at K = 2–7. Similar to Rosenberg‘s analyses including 377 microsatellites [44] and 993 SNPs [24], we found stable results with two clusters anchored by Africa and the Americas at K = 2 (20/20 runs) and a separation of Africa at K = 3 (19/20). At K = 4, a new cluster emerged isolating either the Americas (11/20) or alternatively Central/South Asia (9/20), and at K = 5 both of these regions were isolated (14/20). Most runs separated Europe from the Middle East at K = 6 (17/20), and at K = 7 the main continental regions for whose partitioning the panel was designed were separated from each other in the majority of runs (11/20) and with the highest likelihood.

### Validation of the 41-AIM panel using additional populations of known origin

We further tested the performance of the 41-AIM panel in a realistic setting and estimated the ancestry of 3,077 test subjects from 68 regionally collected populations from the HapMap III and Yale collections. These test populations have been extensively characterized by us and others (see, e.g., [33] and [45]) and are well suited for this purpose. STRUCTURE was run with the HGDP as predefined reference populations at K = 7 (Yale samples were not genotyped for rs2717329). Table 3 shows the average cluster membership of individuals belonging to a specific population for each of the seven continental regions, Africa, the Middle East, Europe, Central/South Asia, East Asia, the Americas and Oceania (*n* = 68 populations). We calculated the percentage of subjects that clustered correctly, using criteria of >85% and >50% cluster membership (MS), respectively.

**Table 3:** Continental ancestry based on STRUCTURE analysis of 68 test populations genotyped on the 41-AIM panel with HGDP subjects included as reference populations

**Population**	**Africa**	**Middle East**	**Europe**	**CS Asia**	**E Asia**	**Americas**	**Oceania**	85% MS*	50% MS*	**Population**	**Africa**	**Middle East**	**Europe**	**CS Asia**	**E Asia**	**Americas**	**Oceania**	85% MS*	50% MS*
**Africa (*n *= 823)**										**C.S. Asia (*n *= 142)**									
MBU	**0.988**	0.002	0.002	0.002	0.002	0.001	0.003	1	1	GIH	0.010	0.056	0.032	**0.811**	0.042	0.026	0.024	0.61	0.93
BIA	**0.988**	0.003	0.002	0.002	0.002	0.001	0.002	1	1	KER	0.010	0.064	0.044	**0.805**	0.044	0.021	0.013	0.50	0.93
IBO	**0.975**	0.005	0.004	0.004	0.004	0.003	0.005	1	1	THT	0.040	0.035	0.026	**0.696**	0.065	0.061	0.078	0.21	0.79
YOR	**0.974**	0.005	0.005	0.005	0.004	0.003	0.004	1	1	KCH	0.010	0.053	0.040	**0.590**	0.209	0.065	0.034	0.19	0.63
YRI	**0.969**	0.006	0.005	0.007	0.005	0.004	0.005	0.99	1	**Siberia (*n *= 47)**									
ZRM	**0.957**	0.010	0.008	0.010	0.006	0.004	0.006	0.95	1	KTY	0.007	0.076	**0.224**	**0.198**	**0.330**	0.145	0.020	0	0.06
LSG	**0.947**	0.008	0.009	0.008	0.007	0.007	0.015	1	1	**East Asia (*n *= 680)**									
LWK	**0.941**	0.017	0.012	0.012	0.007	0.005	0.006	0.95	1	ATL	0.003	0.004	0.005	0.007	**0.965**	0.011	0.005	1	1
HAS	**0.930**	0.020	0.012	0.015	0.010	0.007	0.008	0.86	1	CHB	0.004	0.005	0.005	0.007	**0.963**	0.010	0.007	1	1
CGA	**0.889**	0.036	0.025	0.026	0.010	0.006	0.008	0.71	1	CHS	0.006	0.006	0.005	0.009	**0.960**	0.008	0.005	1	1
MAS	**0.844**	0.060	0.039	0.029	0.011	0.010	0.006	0.45	1	JPN	0.005	0.005	0.004	0.006	**0.967**	0.008	0.005	1	1
SND	**0.841**	0.050	0.030	0.038	0.023	0.009	0.010	0.47	1	CHT	0.006	0.004	0.004	0.007	**0.961**	0.012	0.007	0.98	1
AAM	**0.781**	0.069	0.070	0.039	0.017	0.012	0.012	0.44	0.91	KOR	0.004	0.005	0.004	0.007	**0.964**	0.010	0.006	0.98	1
ASW	**0.761**	0.057	0.092	0.042	0.017	0.023	0.008	0.24	1	AMI	0.005	0.005	0.004	0.008	**0.966**	0.005	0.007	0.97	1
MKK	**0.748**	0.120	0.051	0.055	0.011	0.008	0.007	0.22	0.99	CHD	0.005	0.006	0.006	0.008	**0.957**	0.010	0.008	0.97	1
ETH	**0.430**	0.402	0.096	0.055	0.006	0.004	0.007	0	0.39	HKA	0.004	0.005	0.005	0.008	**0.956**	0.013	0.008	0.97	1
**Middle East (*n *= 165)**										JPT	0.005	0.006	0.005	0.008	**0.957**	0.016	0.005	0.97	1
YMJ	0.012	**0.745**	0.166	0.061	0.007	0.004	0.004	0.47	0.85	LAO	0.012	0.011	0.009	0.015	**0.930**	0.011	0.012	0.91	1
KWT	0.029	**0.685**	0.072	0.158	0.024	0.016	0.016	0.36	0.79	MVF	0.007	0.014	0.012	0.019	**0.924**	0.018	0.006	0.90	1
SAM	0.004	**0.648**	0.269	0.058	0.009	0.009	0.003	0.32	0.76	CBD	0.017	0.034	0.021	0.022	**0.878**	0.009	0.018	0.85	1
DRU-1	0.009	**0.531**	0.353	0.087	0.006	0.005	0.009	0.25	0.53	YAK	0.008	0.017	0.022	0.021	**0.895**	0.031	0.007	0.67	1
**Europe (*n *= 853)**										MLY	0.017	0.024	0.041	0.057	**0.765**	0.014	0.082	0.40	0.90
FIN	0.005	0.036	**0.870**	0.053	0.014	0.015	0.007	0.66	0.97	**Americas (*n *= 298)**									
IRI	0.005	0.099	**0.841**	0.036	0.006	0.006	0.007	0.72	0.92	KAR	0.002	0.001	0.001	0.002	0.003	**0.988**	0.002	1	1
EAM	0.005	0.112	**0.818**	0.046	0.008	0.007	0.005	0.68	0.89	SUR	0.001	0.002	0.002	0.002	0.003	**0.988**	0.002	1	1
DAN	0.007	0.116	**0.811**	0.045	0.006	0.011	0.005	0.69	0.90	PMM	0.005	0.007	0.007	0.011	0.021	**0.943**	0.006	0.95	1
CEU	0.007	0.111	**0.806**	0.055	0.009	0.007	0.005	0.63	0.90	COL-1	0.003	0.007	0.008	0.017	0.011	**0.947**	0.006	0.92	1
RUA	0.005	0.076	**0.798**	0.078	0.016	0.017	0.009	0.50	0.93	TIC	0.008	0.015	0.009	0.033	0.020	**0.904**	0.010	0.73	1
HGR	0.005	0.154	**0.766**	0.051	0.010	0.007	0.006	0.51	0.84	QUE	0.021	0.035	0.040	0.026	0.034	**0.836**	0.009	0.55	1
RUV	0.005	0.130	**0.689**	0.136	0.013	0.016	0.011	0.44	0.76	MAY	0.029	0.041	0.027	0.044	0.028	**0.815**	0.016	0.52	0.97
TSI	0.006	0.271	**0.666**	0.044	0.005	0.005	0.003	0.30	0.71	MUS	0.010	0.094	**0.304**	0.075	0.031	**0.470**	0.015	0.10	0.50
KMZ	0.005	0.064	**0.661**	0.172	0.037	0.038	0.023	0.18	0.80	MEX	0.024	0.179	**0.248**	0.164	0.032	**0.344**	0.009	0	0.26
ASH	0.007	0.329	**0.549**	0.095	0.010	0.006	0.005	0.22	0.58	**Oceania (*n *= 69)**									
ADY	0.005	0.184	**0.540**	0.241	0.016	0.010	0.005	0.22	0.58	PNG-1	0.006	0.003	0.003	0.003	0.009	0.004	**0.972**	1	1
SRD-1	0.006	0.398	**0.538**	0.038	0.009	0.006	0.005	0.30	0.58	NAS	0.006	0.004	0.004	0.005	0.015	0.006	**0.960**	0.92	1
CHV	0.005	0.110	**0.535**	0.224	0.084	0.023	0.019	0.22	0.51	MCR	0.020	0.014	0.008	0.019	**0.680**	0.010	**0.249**	0	0.14
RMJ	0.005	0.490	**0.453**	0.039	0.007	0.003	0.003	0.13	0.46	SMO	0.018	0.031	0.034	0.056	**0.694**	0.016	**0.151**	0	0

*85% MS (50% MS): percent of subjects with at least 85% (or 50%, respectively) membership in the geographically pre-assigned continental cluster.

We found that African populations had very high cluster membership in the African cluster, but East African populations (e.g., Chagga, Maasai and Sandawe) showed slightly lower values. As expected, admixed African Americans as well as a population of Ethiopian Jews showed some cluster membership in Europe and the Middle East, and less than 50% of the subjects were included in the African group at the 85% MS criteria.

The ethnoreligious Samaritans, Yemenite Jews and Druze clustered with the Kuwaiti predominantly in the Middle East, but also showed a significant European contribution. As expected, most European populations clustered predominantly with Europe. However, there was a significant Middle Eastern component, even for the Northern European populations such as the Finns and Irish, demonstrating the somewhat reduced specificity of the 41-AIM panel to distinguish between Europe and the Middle East compared to the resolution between other continents. When applying the less stringent 50% MS criterion, most populations had over 90% of their subjects placed in Europe. Not surprisingly, the Russian populations Adygei, Chuvash, Komi Zyriane and Russian Vologda were found to have a significant Central/South Asian component.

The Central/South Asian cluster included the majority of the Gujarati, Keralite and Thoti Indians at the 50% MS criterion. As expected, the Kachari Assam, located in the East, also showed a significant East Asian contribution. However, there was no predominant placing in any of the seven continental groupings for the Khanty, a population from western Siberia. This is expected since the current continental grouping at K = 7 does not include a specific Siberian/North Asian cluster. The Khanty are currently our only representatives of this large geographic area.

The East Asian test subjects from 15 diverse populations clustered in East Asia with almost no exception. Most Southern Malaysians also showed some Central/South Asian contribution. Most Native American populations clustered predominantly in the Americas. Exceptions were the admixed Muscogee and HapMap Mexicans, which were not placed in this cluster, but showed a strong European component. The Oceanic cluster included all Papua-New Guinean and Nasioi Melanesian subjects. However, the Micronesian and Samoan subjects from this broad geographic area were not assigned to Oceania at the 50% MS criterion, but were found to be admixed with a strong East Asian component.

**Figure 2:**
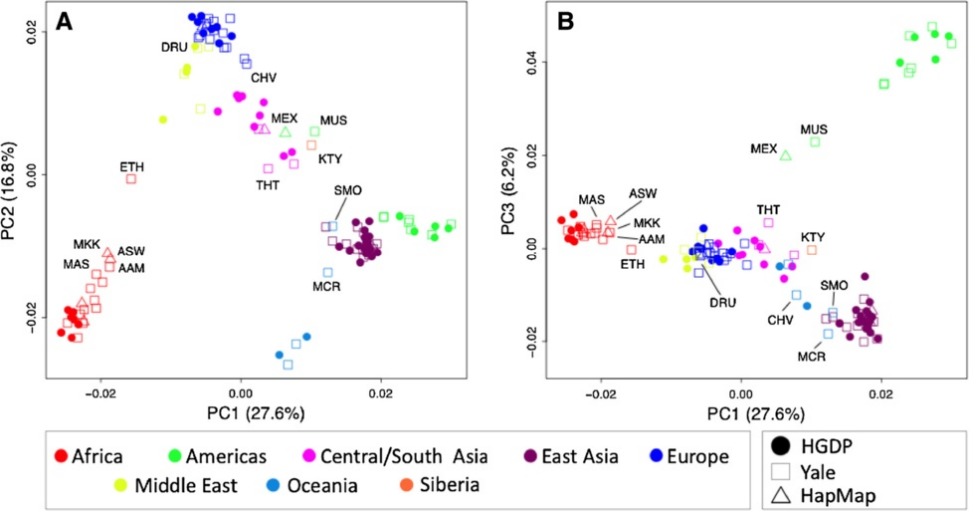
**Principal component analysis (PCA) based on genotype data of 41 AIMs including 4,018 subjects from 120 populations from the HGDP, HapMap and Yale collections.** Individual values of subjects belonging to the same population are averaged to highlight the relative location of specific populations.

Finally, we combined the HapMap III and Yale collections with the HGDP, and further analyses were conducted with our complete reference population set including 4,018 subjects genotyped on the 41-AIM panel. A principal component analysis (PCA) including all 4,018 subjects and averaged for each of the 120 populations is shown in Figure 2. We found that the first PC explained 27.6% of the genetic variability in the data set and corresponded with the Africa to Americas gradient found by STRUCTURE at K = 2. PC2 explained an additional 16.8% variability and added a European component (Panel A). Of note is the misleading positioning of the admixed HapMap Mexicans (MEX) and Native American Muscogee (MUS), both falling within the East Asian cluster. Adding PC3, which accounted for another 6.2% of the genetic variability and includes the Native American component, resolved the structure and correctly placed the MEX and MUS between Europe and the Americas (Panel B).

**Figure 3:**
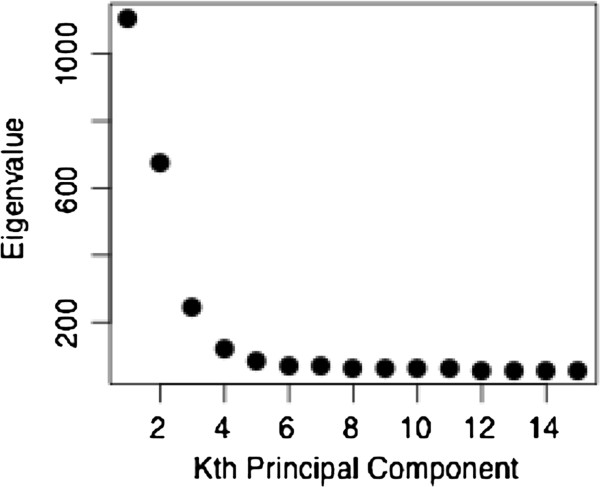
Eigenvalues of the first 15 principal components (PCs) indicating that most genetic variation among the seven continental regions captured by the 41-AIM panel is accounted for by the first 5 PCs.

We performed an analysis of the eigenvalues of the first 15 PCs and found that over 56% of the genetic variation among the seven continental regions was accounted for by the first five PCs (Figure 3).

### Applications of the 41-AIM panel

To highlight a practical application of the 41-AIM panel and our large collection of reference populations, we considered the case of a genetic association study with subjects collected in Southern California. In order to minimize spurious results due to population stratification (i.e., false-positive associations between a phenotype and genetic marker), a PCA is often applied. PCs can be used as an easy tool to visualize large amounts of data or can be included as covariates in association analyses to adjust for population stratification.

**Figure 4:**
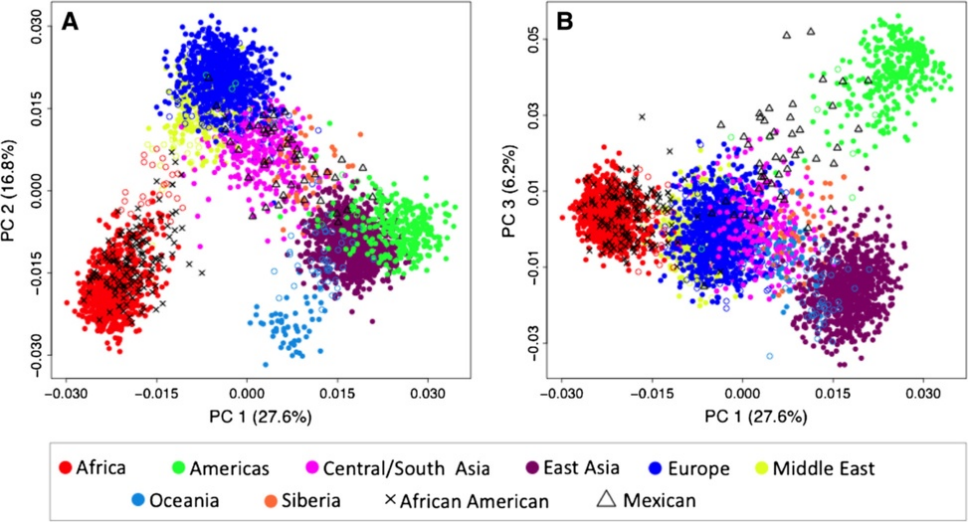
**PC plots of 4,018 reference subjects based on genotype data from the 41-AIM panel.** Subjects are color coded according the geographic sample origin. Admixed populations, African Americans and Mexicans are indicated by open symbols (see text). The % of variation explained ranges from 27.6% for PC1 to 6.2% for PC3.

PC plots of the first three PCs generated based on genotype data of the 41 AIMs are shown in Figure 4 for the complete reference set of 4,018 subjects from 120 populations. When placed in the context of clusters, several populations appear as admixed among the eight colored continental regions (see Table 3; Siberia has been added as its own region here) or are truly admixed (such as the African Americans, Mexicans, Mozabites and others). To increase resolution, we removed these populations (*n* = 13, open symbols) in specific applications.

**Figure 5:**
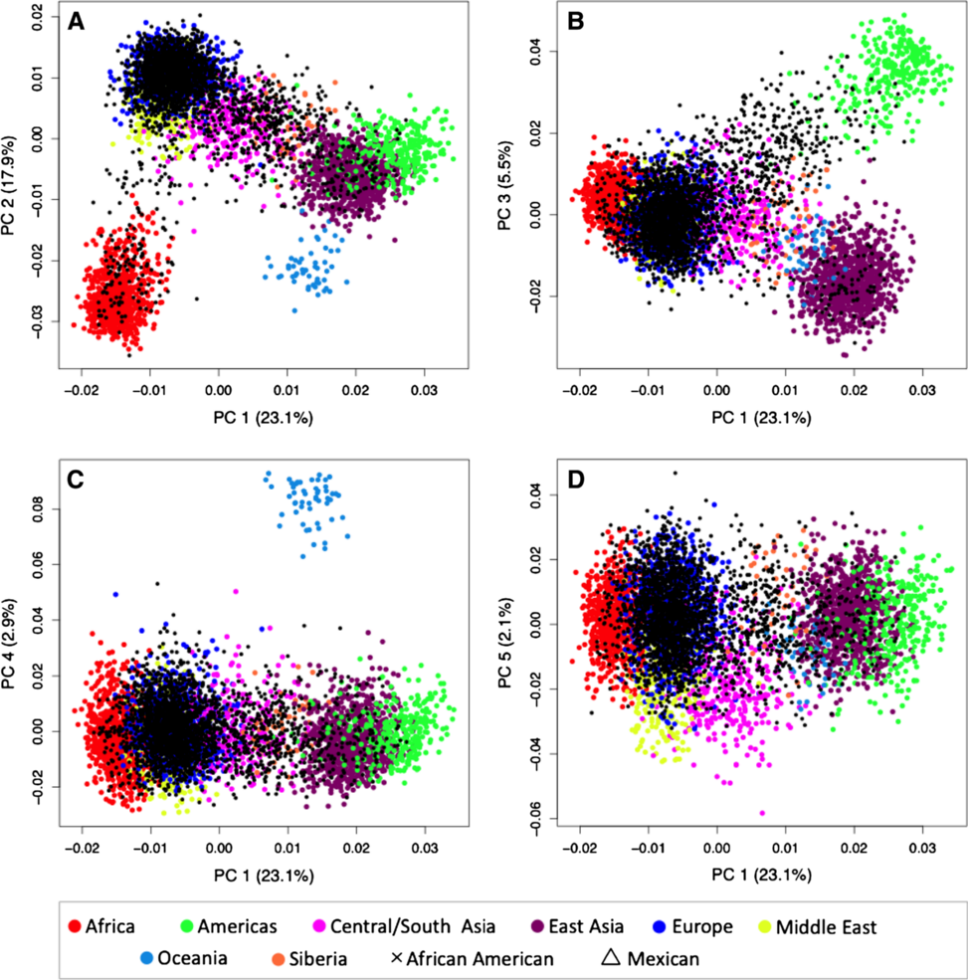
**PC plots of the first five PCs for a visual inspection of a large population sample collected in Southern California (*black*).** Subjects from 107 typical reference populations are color coded. The % of variation explained is indicated in parentheses for each PC.

Figure 5 shows PC plots of Southern Californian test subjects and 107 ‘typical’ reference populations. The first five PCs (PC1 - PC5) explain a total of 51.5% variability in the data, and each identifies different aspects of the population distribution. PC2 highlights the European-African gradient and identified African Americans (panel A), PC3 added the native American component and separated Mexican Americans from Central/South Asians (panel B), and PC4 separated Oceania (panel C). Corresponding to the small eigenvalue of the fifth PC (see Figure 3), PC5 explained only a small fraction of the genetic variability (2.1%) in this setting and did not lead to a strong separation of the eight geographic clusters (panel D). However, PC5 was found to show a North–south cline in Eurasian populations, as indicated by a significant correlation of PC5 values with the average latitude of 77 Eurasian populations (Spearman’s ρ = 0.62, *p* < 0.001). An often-applied alternative to the PCA is the multidimensional scaling (MDS) approach implemented in the genetic association software PLINK. MDS analyses lead to essentially the same results (Additional file 3: Figure S1).

Guided by these visual approaches, subjects are then typically grouped into a small number of more homogeneous groups (e.g., European Americans or African Americans) prior to association analysis, using clustering methods such as implemented in STRUCTURE. Additional population stratification and varying degrees of individual admixture are then accounted for within these more homogeneous groups.

**Figure 6:**
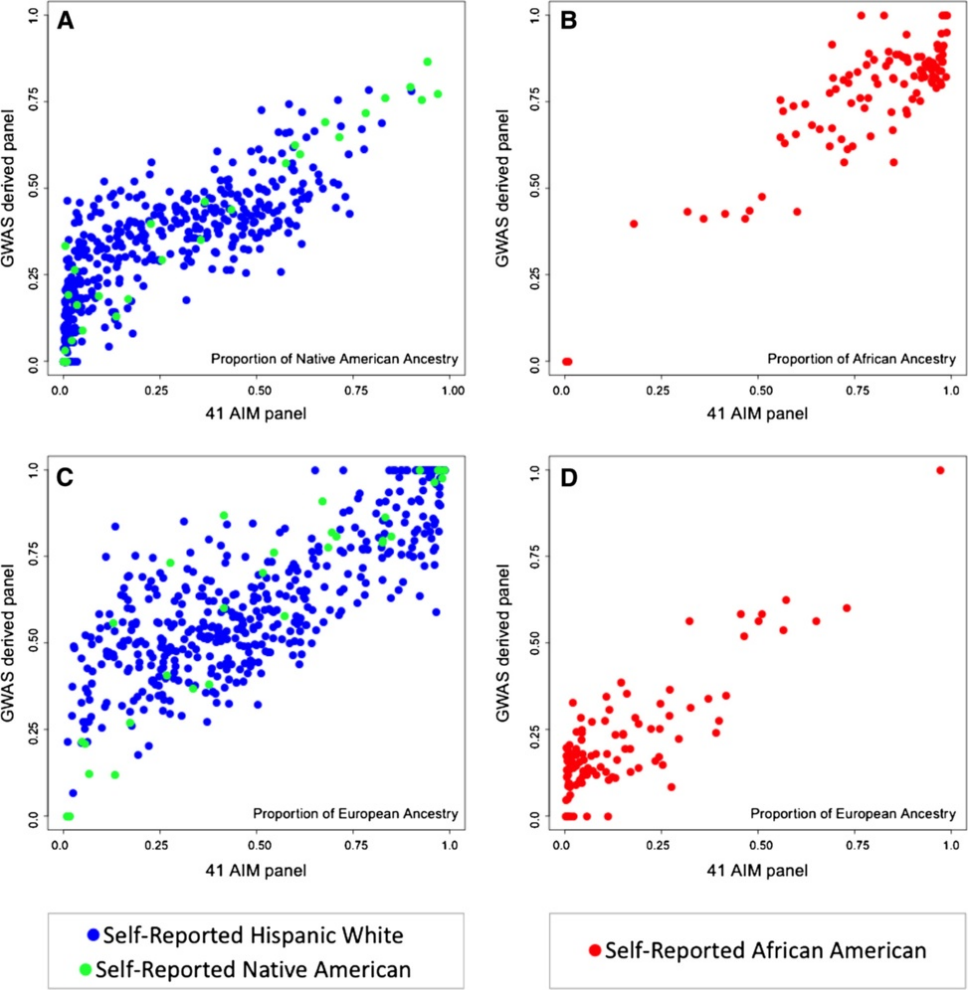
**Comparison of individual admixture estimates based on a large GWAS-derived marker panel and the 41-AIM panel in admixed populations collected in Southern California.** Self-identified Hispanic-White and Native American subjects show a wide range in the degree of Native American and European ancestry proportions (panels **A** and **C**). Self-identified African Americans show a range in the degree of African and European ancestry proportions (panels **B** and **D**).

To assess the informativeness of the AIM panels in detecting admixture at the individual level, we compared STRUCTURE admixture estimates based on the 41 and 31 AIMs with independently derived estimates based on a large, GWAS-derived panel (see Methods). Subjects were selected based on self-report from the admixed African American and Hispanic White and Native American populations (Figure 6). Individual ancestry proportions derived from the 41-AIM and GWAS panels were strongly correlated for both the Hispanic White and Native American populations (*n* = 484, Pearson’s *r* = 0.81 for the proportion of Native American ancestry in panel A, *r* = 0.81 for the proportion of European ancestry in panel C) and the African Americans (*n* = 106, Pearson’s *r* = 0.86 for the proportion of African ancestry in panel B, *r* = 0.85 for the proportion of European ancestry in panel D; all *p* < 2 × 10^-16^). Slightly reduced correlations of individual admixture estimates were achieved between the GWAS-derived and smaller 31-AIM panels (A: *r* = 0.77, B: *r* = 0.78, C: *r* = 0.84 and D: *r* = 0.85, respectively). Importantly, a detailed inspection of the scatter plots indicates that the AIM panels lack sensitivity in detecting admixture in individuals with low proportions of admixture (in the range of < 20-25%) when compared to the GWAS-derived panel.

## Discussion

### Application and limitations

Our motivation was to develop a feasible method to discern continental ancestry that would enable a safeguard against the impact of population stratification in small genetic association studies where limited resources preclude large genotyping efforts. We achieved this by choosing a very small set of highly discriminative AIMs suitable for multiplexing applications, thus enabling lower cost and higher throughput. To ensure a wide application potential, we optimized our panel for two commonly used multiplex platforms, the ABI SNPlex [25] and Sequenome iPLEX systems [26]. At the same time, these AIMs perform well in single SNP TaqMan assays and can also be extracted from whole-genome arrays such as the Illumina HumanHap550 chip, thus allowing an easy combination of samples with genotyping from different sources. This is especially important for AIMs, where imputation of SNPs based on information from genotyped markers is not advisable.

Our panel was able to accurately discern the global ancestry of a large majority of subjects originating from one of the seven specific ancestral clusters. This was the case for both the full 41-AIM panel and the subset of 31 AIMs, indicating that a balanced reduction of markers in these small panels did not significantly impact the robustness of the results. A direct comparison of our findings with a previously published small panel of 93 AIMs published by Seldin’s group [10] showed 89.7% agreement in continental assignment of HGDP subjects (data not shown), further validating our panel.

Not surprisingly, the biggest limitation to imputing global ancestry was found for subjects from Eurasia, where low F_ST_ values of 0.06 - 0.09 among Europe, the Middle East and Central/South Asia indicated little genetic diversity. The clinal distributions of allele frequencies between Europe and East Asia pose a challenge for the identification of highly discriminative markers, a limitation also impacting other small AIM panels [12,46]. We therefore suggest supplementing our panel with additional high-resolution markers for studies with focus on Eurasia. Such markers suitable for discerning specific pairs of regions can easily be extracted from our extensive preselected list of global AIMs.

### Impact of reference populations

Independent of the statistical method used to determine ancestry and admixture proportions, the results of these analyses depend not only on the informativeness of the genetic markers, but also strongly on the set of reference populations included. An omission of reference subjects from an ancestral group likely leads to misclassification of test subjects with similar ancestries. For example, we previously found that African Americans clustered strongly with Central Asians in a three-way admixture analysis (erroneously) including only reference subjects from Europe, Central Asia and the Americas.

We considered this crucial issue during panel development and leveraged the publicly available 52 HGDP populations collected across the globe [47]. We then increased our reference set by leveraging AIM frequencies from the HapMap III and performing additional AIM genotyping in our large global collection [33]. With over 4,000 subjects from 120 global populations, we thus assembled one of the largest reference sets published for the purpose of ancestry determination. However, specific regions such as Siberia are still underrepresented, and efforts to expand our reference subject collection are ongoing.

### Admixed subjects

Whereas ancestry assignment of subjects from a specific geographic area represented by a cluster in the reference population set is a quantifiable and relatively straightforward task, admixed subjects resulting from ancient or recent contact of populations with distinct ancestries pose challenges.

If such a cohort consists of admixed subjects with known ancestry contributions, such as two-way admixed Mexican Americans collected from a distinct area in Southern California, the varying degree of European and Native American ancestries can easily be estimated in admixture analyses implemented in Statistical packages such as STRUCTURE, ADMIXTURE [43] or BAPS [48]. Our AIM panels were able to detect admixture in individuals from these populations, but as expected for such small panels, were less sensitive when individual admixture proportions were low.

However, in a clinical sample including subjects of unknown ancestral origin and complex population structure, as is often the case in our studies (e.g., [35,49,50]), the presented methods may lack specificity to distinguish between admixture of distinct populations and erroneously place admixed subjects together with intermediate populations. This is especially true when including only the first few components of multivariate data reduction methods such as MDS and PC analyses (see, e.g., Figure 2, Panel A, for Mexican Americans clustering with Central/South Asians). In these cases, adding demographic information such as self-declared race and ethnicity information is strongly suggested to help minimize misassignments.

### Challenges for genetic association studies

Adding to the complexities of accurately differentiating ancestral groups and estimating admixture proportions is the appropriate incorporation of this information into the design of genetic association studies. While the negative impact of population structure on association studies is well known [6], and methods to control for it are established and now routinely applied to studies of relatively homogeneous cohorts such as typically collected for GWAS (see e.g. a recent review [5]), the situation remains challenging for heterogeneous clinical collections or epidemiological cohorts.

Depending on the composition and relative numbers of subjects from different ancestral backgrounds, common questions in such studies include the genetic definition of African Americans, which typically show degrees of European admixture that vary among individuals [51]. There is currently no consensus for an appropriate cutoff point between European Americans and African Americans. Even less trivial is the incorporation into association studies of three-way admixed subjects such as Caribbean Latinos originating from Puerto Rico and the Dominican Republic [52], typically showing both Native American and high levels of African ancestry.

For practical purposes, we often employ a multi-tier approach: we first group subjects into continental clusters using a majority criterion with statistical methods such as STRUCTURE and then confirm the plausibility of the grouping with demographic data, where available. Next, we aim to place most subjects into a very small number of clusters including genetically similar subjects, for example, by combining similar continental groups such as from Eurasia, and excluding outliers of minority ancestries. Lastly, we control for additional population stratification within clusters by incorporating MDS components into association studies and ultimately combine results in meta-analyses, where appropriate. Such a method was, for example, employed for the Southern Californian population sample presented here, which encompassed a wide array of self-declared ethnic groups. Our approach resulted in a four-cluster analysis with 61% European Americans, 18% subjects with Native American admixture, 7% subjects with African admixture, and 15% subjects of other ancestry and/or complex admixtures.

## Conclusion

In conclusion, we demonstrated the utility and limitations of a small AIM panel specifically developed to discern global ancestry. We believe that it will find wide application because of its feasibility and potential for a wide range of applications. To allow this reference set to be readily accessible for others to use, we are entering the allele frequencies for these 41 SNPs into ALFRED (alfred.med.yale.edu) [34] as an “SNP Set.” To allow ready estimation of likelihoods of ancestry of individuals, these SNPs are also being entered as an additional AISNP Panel in FROG-kb (frog.med.yale.edu) [53].

## Abbreviations

ALFRED: allele frequency database; AIM: ancestry informative marker; AISNP: ancestry informative single nucleotide polymorphism; δ: allele frequency differences; MS: cluster membership; GWAS: genome-wide association study; I_n: marker informativeness; MDS: multi-dimensional scaling; PC: principal component; PCA: principal component analysis.

## Competing interests

The authors declare that they have no competing interests.

## Author‘s contributions

All authors read and approved the final manuscript. CMN conceived of the study, participated in its design and coordination, and wrote the manuscript. TS carried out genotyping. AXM performed the statistical analyses. KKK and JRK participated in the coordination of the study and in the writing of the manuscript. XW carried out genotyping and assisted in preliminary analyses at Yale. All authors read and approved the final manuscript.

## Supplementary Material

Additional file 1: Table S1 Geographic sampling location, population name, number of subjects and source of genotype data of 120 reference populations. Click here for file

Additional file 2: Table S2 Chromosomal position (GRCh37.p5), alleles and informativeness (I_n) of 1,442 continental AIMs and sequence information for the multiplex 41-AIM and 31-AIM panels. Click here for file

Additional file 3: Figure S1 MDS plots of the first five MDS components for a visual inspection of a large population sample collected in Southern California (*black*). Subjects from 107 typical reference populations are color coded. Click here for file
