# Supplementary material for: Inference of human continental origin and admixture proportions using a highly discriminative ancestry informative 41-SNP panel
Source: Investig Genet. 2013 Jul 1;4:13. doi: 10.1186/2041-2223-4-13 (PMC3699392; doi:10.1186/2041-2223-4-13)
Supplement: Additional file 1: Table S1 — Geographic sampling location, population name, number of subjects and source of genotype data of 120 reference populations. [file 2041-2223-4-13-S1.docx]

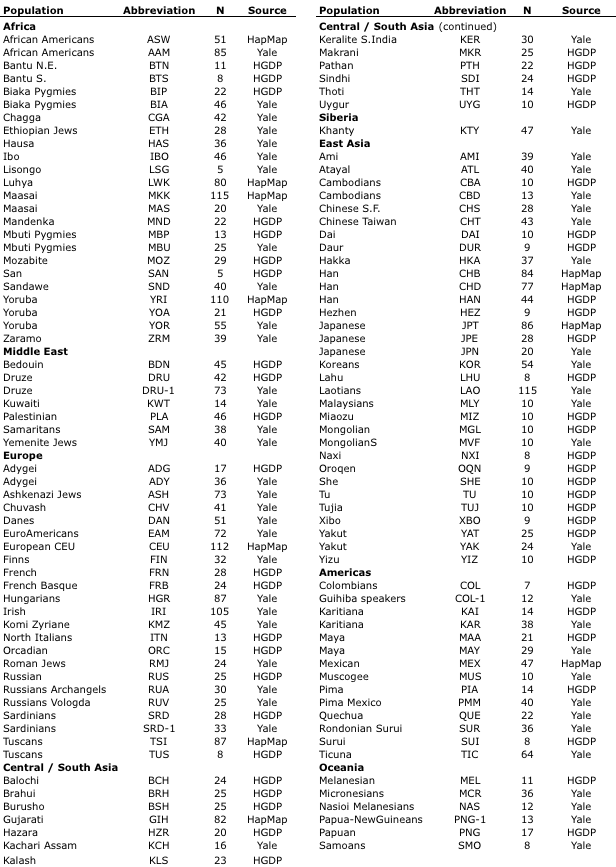
Additional Table 1: Geographic sampling location, population name, number of subjects and source of genotype data of 120 reference populations
