# Supplementary figures and images for: Inference of human continental origin and admixture proportions using a highly discriminative ancestry informative 41-SNP panel
Source: Investig Genet. 2013 Jul 1;4:13. doi: 10.1186/2041-2223-4-13 (PMC3699392; doi:10.1186/2041-2223-4-13)

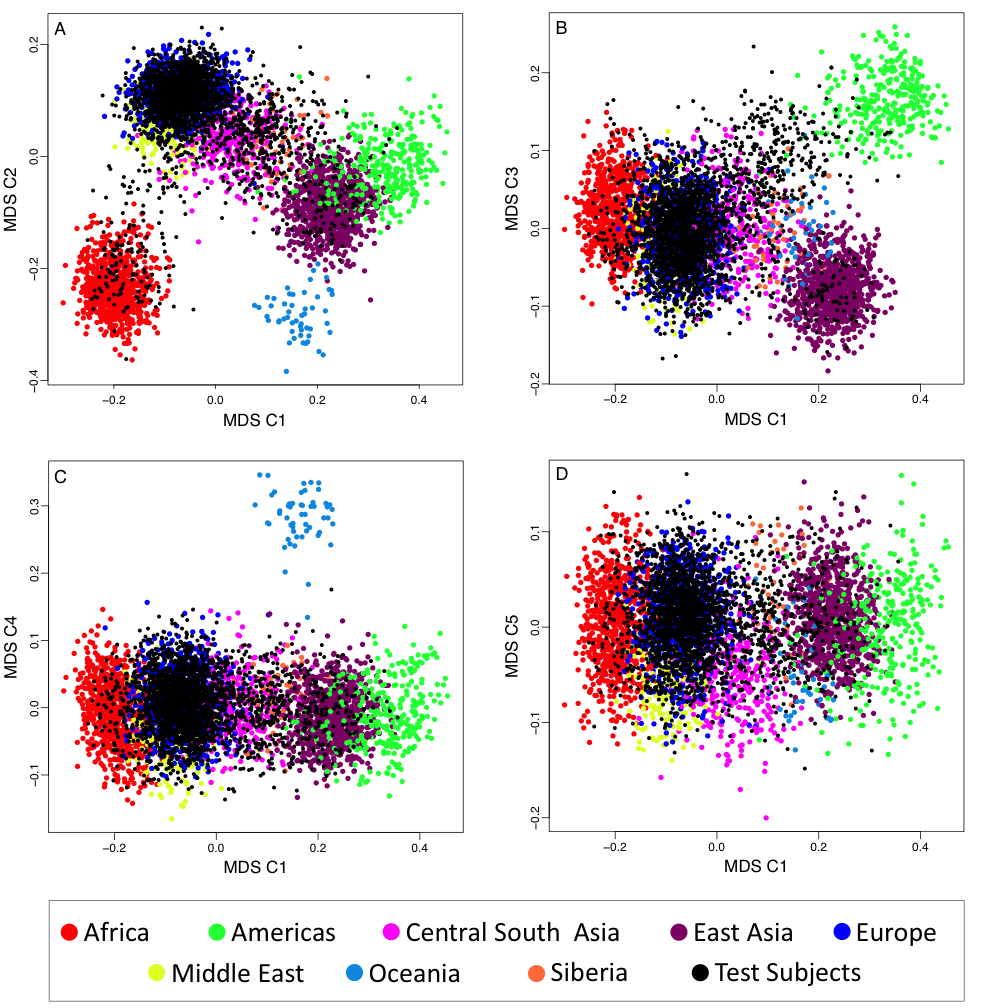

Supplement: Additional file 3: Figure S1 — MDS plots of the first five MDS components for a visual inspection of a large population sample collected in Southern California (black). Subjects from 107 typical reference populations are color coded. [file 2041-2223-4-13-S3.docx]
